# Analysis of Regulatory Botanical Submission Profile for Cancer Management from the U.S. FDA Perspectives

**DOI:** 10.1007/s43441-025-00786-y

**Published:** 2025-06-04

**Authors:** Jin-Young K. Park, Daniel Lee, Lixin Rui, Xiaoyue Gao, M. Scott Furness, Charles Wu

**Affiliations:** 1 Office of Pre-Market Additive Safety, Office of Food Chemical Safety, Dietary Supplements, and Innovation, Human Foods Program, Food and Drug Administration, College Park, MD 20740 USA; 2 Office of Oncologic Disease, Office of New Drugs, Center for Drug Evaluation and Research, Food and Drug Administration, Silver Spring, MD 20993 USA; 3 Department of Medicine, School of Medicine & Public Health, University of Wisconsin-Madison, 4053, WIMR, 1111 Highland Ave, Madison, WI 53705-2275 USA; 4 Office of New Drug Products, Office of Pharmaceutical Quality, Center for Drug Evaluation and Research, Food and Drug Administration, Silver Spring, MD 20993 USA; 5 WO52 RM 4136, HFD-850, 10903 New Hampshire Ave, Silver Spring, MD 20993-0002 USA

**Keywords:** FDA/CDER botanical submission, Cancer management, Drug development, Oncological application, Regulatory experience

## Abstract

**Background:**

The United States Food and Drug Administration (FDA) has received over 700 botanical investigational new drug applications (INDs) in a broad spectrum of therapeutic areas since 1984. The greatest numbers were for cancer management. The aims of our study were to conduct a first-time, in-depth analysis of the regulatory submission profiles for botanical INDs with oncologic indications, in comparison with non-oncologic indications, and to share our regulatory review experience of oncologic botanical drug research and development.

**Methods:**

The FDA Center for Drug Evaluation and Research (CDER) maintains an in-house database of botanical INDs that contains many data elements, including initial 30-day actions (safe-to-proceed, clinical hold, etc.), current regulatory status, primary purpose of the proposed clinical trials, and initially proposed clinical trial phase information by sponsor. The database provided internally validated regulatory submission information that FDA received between March 1984 and December 2020 for 254 botanical INDs with oncologic indications, as well as 485 non-oncologic botanical INDs.

**Results:**

A higher percentage of the oncologic botanical INDs (69% versus 58% for non-oncologic botanical INDs, *p* < 0.01) received an initial 30-day safe-to-proceed designation to initiate the clinical investigations. One hundred thirty-seven oncologic botanical INDs were submitted to conduct phase 1 trials to investigate the safety and tolerability of their products, and 46 of these INDs are currently active. An additional 117 INDs were proposed to conduct phase 2 or phase 3 trials to assess safety and efficacy of oncologic botanical products, and 36 of those INDs are currently active, including 3 INDs in phase 3 trials. Most of the oncologic botanical INDs were for the investigation of specific solid tumors (71%) with more than one third of these related to prostate and breast tumors.

**Conclusions:**

Despite the scientific and regulatory challenges that FDA reviewers previously experienced, our analysis shows that there were over 80 currently active botanical oncologic INDs, including several in the late phase of drug development for cancer management. The implication of this finding is significant in that many clinical trials of botanical drug products intended to provide high-quality cancer patient care are in the regulatory pipeline.

## Introduction

Cancer is a major public health concern in the United States with 1.9 million new cancer cases and 609,820 cancer-related deaths projected to occur in 2023 [1]. Although advances such as targeted therapy with small molecules, monoclonal antibodies, immunotherapy and precision oncology have shown promising results, serious side effects and resistance to therapy present continuous challenges [2]. Cancer patients also suffer from long-term side effects of cancer treatment, including fatigue, sleep disorder, nerve damage, and pain. Development of complementary and alternative pharmacological approaches has the potential to help prevent, suppress, or reverse the initial phases of carcinogenesis [3]. Many cancer patients reported using herbal- and other natural remedies to treat late and long-term effects [4, 5]. Thus, the complementary and alternative pharmacological approaches can treat cancers with reduced side-effects. However, human safety and labeling drug claims of most herbal products and dietary supplements are not evaluated by FDA [6]. Predicting clinical effects of these products is often challenging due to a lack of human data from well-designed clinical trials.

The U.S. Food, Drug, and Cosmetic (FD&C) Act requires that clinical investigations involving unapproved drugs be submitted for FDA’s review under Investigational New Drug Applications (INDs) [7]. The FDA has been receiving botanical INDs since 1984 for a variety of indications [8]. The FDA faced various regulatory and scientific challenges while reviewing an increased number of botanical submissions (i.e., from two submissions in 1984 to over forty submissions in 2020). For instances, heterogeneous complexity of botanicals, particularly in multiple-herb products, and the active components that are not fully identified, presents challenges in characterizing their pharmacology, ensuring quality consistency, and demonstrating therapeutic efficacy. Due to the significant variations in the chemical composition and bio-pharmacological activity even from the same botanical source, conventional chemistry, manufacturing, and controls (CMC) approaches used for quality control of small, single-molecule products alone sometimes are not sufficient. Therefore, in 2016, FDA published a Guidance for Industry for the Development of Botanical Drugs, which defined the term *botanicals* as any complex “products from plant materials, algae, macroscopic fungi, and combinations thereof,” [9] and provided recommendations to consider during early and late-phase trials to facilitate the drug development of their products and ultimately, to ensure the batch-to-batch consistency. In 2020, FDA also published a report on an overview of the botanical INDs received in the years preceding 2018 [10], and discussed their regulatory experience and scientific challenges in the review of botanicals intended to be used as drugs. Interestingly, the report noted that among the various therapeutic areas, cancer and related conditions comprised the highest percentage of botanical IND submissions (i.e., 33% versus less than 11% in all other therapeutic areas) [10]. Currently, there are four successful examples of approved botanical drugs, Veregen in 2006 [11], Fulyzaq in 2012 [12], NexoBrid in 2022 [13], and most recent approval of Filsuvez in 2023 [14], although all of these were submitted for non-oncologic indications.

The aim of our work was to conduct a first-time, in-depth analysis of the regulatory submission profiles of 254 botanical INDs with oncologic indications that FDA received between March 1984 and December 2020, and share the unique regulatory review experience in oncologic botanical drug research and development. Recognizing the scientific and regulatory challenges, FDA takes a holistic approach to bring up more promising active oncologic botanical INDs in the regulatory pipeline leading to safer and more effective botanical drugs in the treatment of cancer This analysis did not include submission data from 2021 until the present because they were still under FDA assessment, and therefore, are beyond the scope of this analysis.

## Methods

The FDA Botanical Review Team (BRT) in the Center for Drug Evaluation and Research (CDER) maintains an in-house database of botanical INDs that spans the period from 1984 (when the botanical IND process began) to the present [10]. As stated above, the data discussed in this article apply to INDs received from March 1984 to December 2020. For the present analysis, we defined oncologic botanicals as botanical IND products with any oncologic or cancer indications which the FDA botanical reviewers or BRT reviewed. In general, the primary divisions responsible for review of the oncologic botanical INDs within the FDA/CDER are Hematology and Oncology Divisions located with the Office of Oncologic Diseases. We designated botanical IND submissions primarily reviewed by these divisions as oncologic botanical INDs. In comparison, the non-oncologic botanical INDs were designated as any indications other than cancer management. Moreover, any synthetic, semi-synthetic or otherwise highly purified compounds were not included in the analysis because they were not defined as botanical drugs [15]. 

The BRT database contains many data elements, including proposed product name, the nature of the application (commercial or research), CDER therapeutic review division, initial 30-day actions (safe-to-proceed, clinical hold, etc.), and current regulatory status (active, withdrawn, hold, etc.). It also includes, if available, information on primary purpose of the proposed drug (e.g., basic research, prevention, treatment, supportive care (palliation), diagnostic application, etc. see ClinicalTrials.gov protocol registration data element [16]), initially proposed clinical trial phase information (phase 1, 2 or 3), the origin of the botanical raw materials (BRM), the number of plants in the BRM, and whether the botanicals have any ingredients used in commercially available dietary supplements or traditional herbal medicinal products in the US.

Pre-submission INDs (PINDs) in our analysis were excluded to avoid potential duplications. Each botanical IND submission (*N* = 739) represents a unique trial phase and regulatory profile. We used STATA Statistics and Data Analysis software [17] for the summary statistics shown in the tables and figures in this report. To determine if there was any difference in the overall 30-day action and regulatory status between the two groups (i.e., 254 botanical oncologic INDs and 485 botanical non-oncologic INDs 485), we used the Chi-square test and calculated overall p-values. We applied Chi-square and Fisher’s exact tests to compare the two groups for individual 30-day actions and regulatory statuses. To adjust for multiple comparisons within each group, we applied False Discovery Rate (FDR) and Bonferroni corrections and calculated individual p-values. A p-value of < 0.05 was considered statistically significant to identify any between-group differences.

## Results

Of all botanical INDs received by FDA through 2020, a large portion (254 out of 739, 34%) was submitted to develop oncologic drugs (commercial application) or for scientific research (research application) to investigate if botanical products were safe and beneficial in the management of cancer (Table 1). A significantly higher percentage of oncologic botanical INDs received an initial 30-day safe-to-proceed (STP) designation, which allowed the initiation of clinical investigations, compared to the non-oncologic botanical INDs receiving STP during the same period (69% versus 58%, *p* < 0.05). Some botanical INDs were initially placed on clinical hold or withdrawn due to insufficient information or concerns regarding the safety of the products, and the FDA actions could delay a proposed clinical investigation or suspend all or some of the ongoing investigations. The percentages of submissions receiving the FDA’s initial hold or withdrawal actions were similar between botanical INDs with oncologic indications and botanical INDs with non-oncologic indications (26% versus 25%). In contrast, the percentages of submissions for the rest of the Agency’s initial actions, including information requests and exemption for IND (under “Others” in Table 1), were significantly less for oncologic botanical INDs than that for non-oncologic botanical INDs (5% versus 17%, *p* < 0.001).

**Table 1:** The FDA’s initial 30-day actions and the current regulatory status for botanical INDs FDA received between 1984 and 2020

		Oncologic Indications (34% of 739 INDs)	Non-Oncologic Indications (66% of 739 INDs)	*p*-value^@^
Initial 30-Day Actions (Overall p-value < 0.01*)	Safe-to-Proceed (STP)	175 (69%)	282 (58%)	< 0.01
	Hold	56 (22%)	111 (23%)	> 0.5
	Withdrawal	12 (5%)	14 (3%)	> 0.1
	Others ^a^	11 (4%)	78 (16%)	< 0.001
	Total	254 (100%)	485 (100%)	
Current Regulatory Status ^#^ (Overall p-value < 0.1)	Active	82 (32%)	171 (35%)	> 0.5
	Hold	7 (3%)	33 (7%)	< 0.05
	Withdrawn or terminated	137 (54%)	237 (49%)	> 0.1
	Inactive	24 (9%)	31 (6%)	> 0.1
	Others ^b^	4 (2%)	13 (3%)	> 0.5
	Total	254 (100%)	485 (100%)	

Superscript “a” included other 89 INDs with inadequately defined initial 30-day action such as Information Requests (IR) and exempt INDs, and other regulatory terms used

Superscript “b” included canceled INDs, undefined, exempt INDs, and INDs with pending status

# The information was current as of December 2020. The definition of each IND status is available at the FDA’s “IND Application Procedures: Investigator’s Responsibilities” at https://www.fda.gov/drugs/investigational-new-drug-ind-application/ind-application-procedures-investigators-responsibilities. The 21 Code of Federal Regulation (CFR) Sect. 312.44 also provides information on the grounds for termination of an IND application. The IND application procedures for “clinical hold” is available at https://www.fda.gov/drugs/investigational-new-drug-ind-application/ind-application-procedures-clinical-hold

^**@**^ P-values were shown as unadjusted before multiple comparisons by applying both FDR and Bonferroni corrections, which were same as adjusted ones except for Hold

* P-value of < 0.05 considered statistically significant

As of December 2020, one third of the botanical INDs with either oncologic indications (82 out of 254, 32%) or non-oncologic indications (171 out of 485, 35%) were active (Table 1) because clinical trials for these INDs were ongoing after the submitters provided adequate responses to the FDA’s information requests (e.g., supportive clinical protocols to assess both the safety and efficacy and/or to lift the clinical hold status). The analysis of the current (as of December 2020) regulatory status indicated that 3% (7 out of 254) of the oncologic IND submissions were on hold, even if they initially received the STP designation. This percentage was lower than 7% (33 out of 485) of non-botanical INDs on hold (Table 1). However, this difference was not statistically significant after adjusting for multiple comparisons (*p* > 0.1). Regardless of the type of submission, about half of all botanical INDs had either been withdrawn by the sponsor or been terminated by the Agency. The termination often occurred when methods, facilities, and controls used for manufacturing the products were inadequate to establish and maintain appropriate standards for quality and purity as needed for patient safety. About 10% of the botanical INDs were inactive, cancelled, exempted, or pending.

**Fig. 1:**
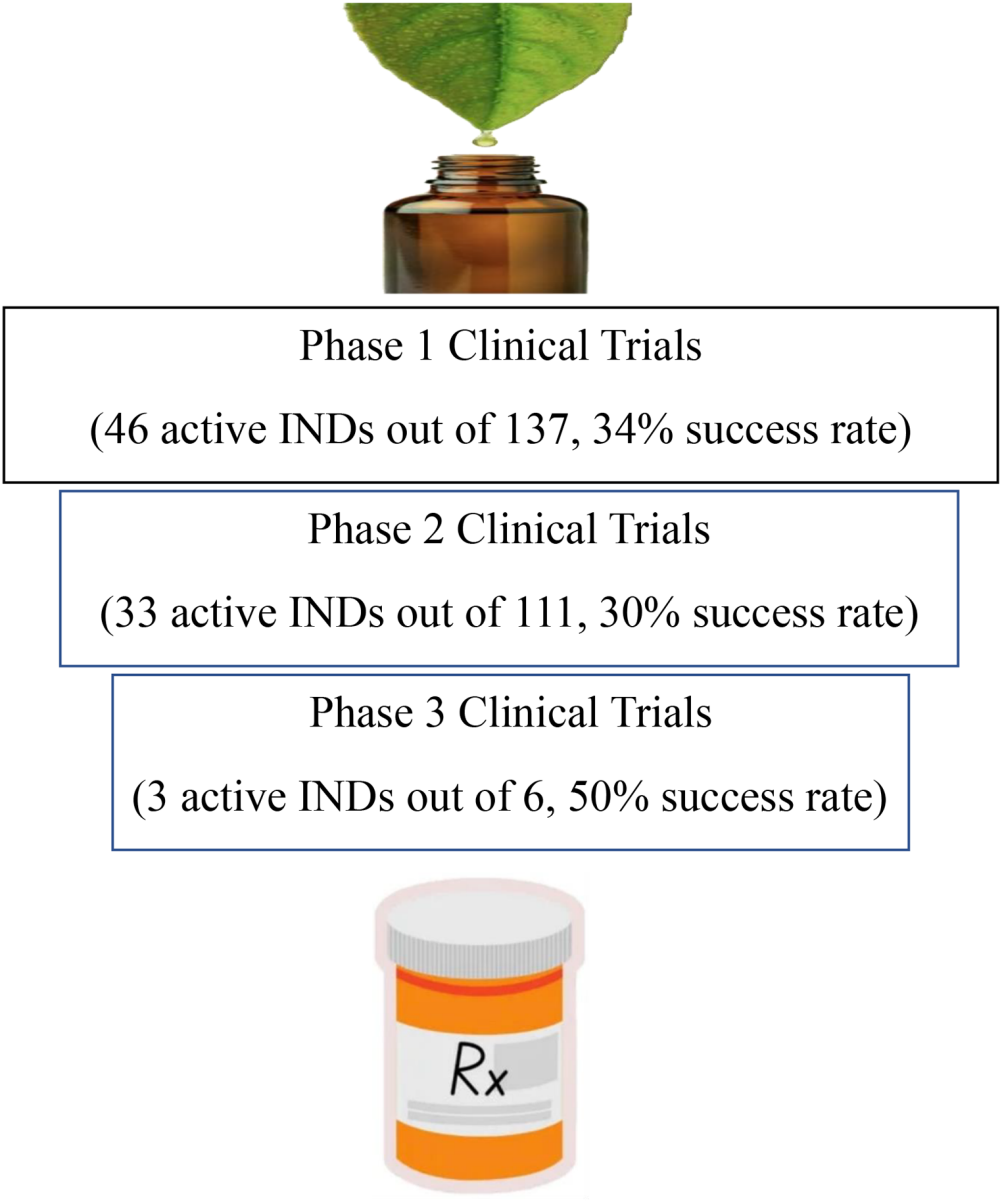
The active botanical INDs with oncologic indication at different clinical trial phases with drug products made from botanical raw materials prior to New Drug Application (NDA) for marketing approval. The “active” status of an IND submission indicates an ongoing clinical investigation as of December 2020, after receiving the Agency’s 30-day initial STP designation. Success rate is defined as the percentage of active (on-going) INDs in each proposed clinical trial phase

**Fig. 2:**
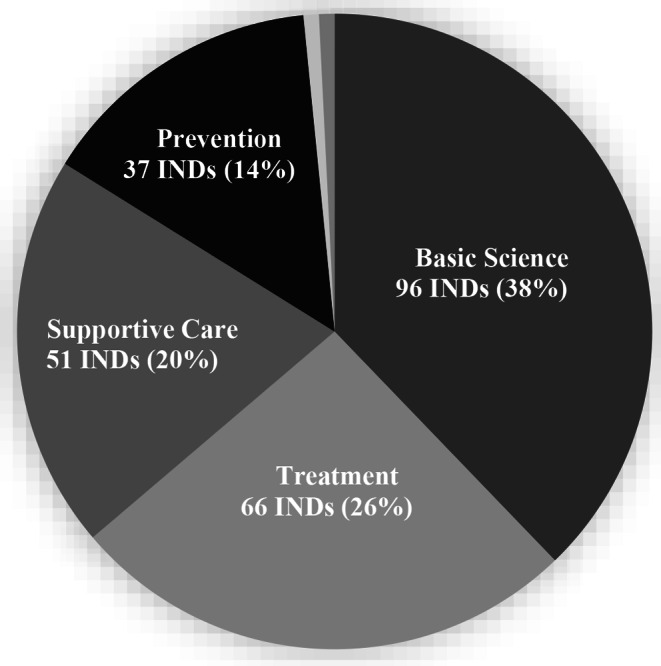
The primary purpose of the clinical trials for the botanical INDs with oncologic indications. (White and light grey areas on top with 2 INDs (1%) each for “diagnostic” and “information not available”)

As shown in Fig. 1, a total of 46 (out of 137, 34%) oncologic INDs that were submitted to conduct phase 1 trials were active as of December 2020. Furthermore, 33 (out of 111, 30%) oncologic INDs were active in phase 2 trials, while 3 (out of 6, 50%) were active in phase 3 trials to assess safety and efficacy. The active 36 oncologic INDs proposing to conduct phase 2 or phase 3 clinical trials address the important goals of prevention of cancer progression, supportive care for cancer patients, and the treatment of cancer. Of all 254 oncologic INDs, more than one third (38%) were submitted for basic research, and the other three major categories were treatment (26%), supportive care (20%), and prevention (14%) (Fig. 2). A few botanical INDs with oncologic indications were submitted for diagnostic purposes, although neither was active.

Analysis of the specific tumor types assessed in botanical INDs with oncologic indications showed that the investigation of specific solid tumors predominantly (181 out of 254, 71%) identified prostate, breast, lung, colon/rectal, and hepatic cancers as the most frequent types (Table 2). In comparison, only 9% (22 out of 254) were indicated for hematologic malignancies, including myeloma, leukemia and other unspecified malignancies, whereas 20% (51 out of 254) of the submissions had no mention of specific tumor types.

**Table 2:** Specific tumor types for botanical INDs with oncologic indications

Specific tumor type			Botanical INDs (%)		
Solid Tumors			181 (71%)		
Hematologic Malignancies			22 (9%)		
Unspecified ^a^			51 (20%)		
Total			254 (100%)		
Solid Tumors	Botanical INDs (%)		Hematologic Malignancies	Botanical INDs (%)	
Prostate	37 (20%)		Myeloma	7 (32%)	
Breast	34 (19%)		Leukemia	5 (23%)	
Unspecified ^a^	20 (11%)		Myelosuppression	2 (9%)	
Colon and/or rectal	14 (8%)		Others ^c^	8 (9%)	
Lung	14 (8%)		Total	22 (100%)	
Hepatic	9 (5%)		c. Other hematologic malignancies included unspecified, B-cell malignancies, hemoglobinopathies, lymphoma, lymphoproliferative syndrome, macroglobulinemia, multiple hematologic malignancies, and myelodysplastic syndrome (1 IND each).		
Ovarian, Pancreatic, or Multiple	7 each (12%)				
Head and neck	6 (3%)				
Others ^b^	26 (14%)				
Total	181 (100%)				

a. No mention of specific tumor types

b. Other solid tumors included polyps, squamous, bladder, oral, leukoplakia, cervical, glioblastoma, melanoma, rectal and brain tumors (2, 6, 4, 3, 2, 2, 2, 2, 2, 1 INDs, respectively)

## Discussion

In this paper, we analyzed the regulatory submission profiles of 254 botanical INDs with oncologic indications and compared the regulatory review experience of oncologic botanical drug research and development with non-oncologic botanical drugs received at the same period. Despite the challenges associated with reviewing complex botanical drug products, our analysis, while maintaining confidentiality of trial products, provides insights into regulatory hurdles and updates the status of promising active (on-going) oncologic botanical IND submissions within the regulatory pipeline.

## Regulatory Action and Status for Developing a New Botanical Drug in the U.S

An IND should be submitted to FDA if a drug not previously authorized for marketing in the US is to be used for clinical investigation or, in certain cases, for the purpose of clinical treatment when no approved therapies are available [18, 19]. An IND proceeds with a clinical investigation once the sponsor has been notified by the FDA that the investigation may proceed (known as an initial 30-day STP) or after 30 days if the IND is not placed on clinical hold [20]. In general, an IND should contain sufficient information to demonstrate that the proposed drug is safe for testing in humans and that the clinical protocol(s) is properly designed for its intended objectives (e.g., efficacy). While these requirements are applicable to botanical drug INDs, FDA recognizes that identification of biologically active substances in heterogeneous botanical mixtures is challenging. FDA’s botanical drug guidance recommends that if proposed botanicals have been used in humans prior to submission with safety data, the sponsor should specifically describe that prior use in the IND submission [9]. FDA has also taken a practical approach with some quality control flexibility for botanical drug products, as well as the fixed-dose combination rule [21]. 

FDA previously reported that approximately one-third of all the botanical IND submissions were received for treatment or management of various cancers [10]. The current analysis showed that the majority (69% as shown in Table 1) of botanical INDs with oncologic indications received STP, which was significantly higher than 58% for non-oncologic INDs receiving STP in the same time period. The high percentage of STP designations for oncologic botanical INDs may reflect differences in regulatory decision-making. Oncologic INDs often allow more flexibility due to the urgent nature of terminal diseases compared to non-oncologic indications. This flexibility ultimately shortens the drug development timeline, aligning with the FDA’s initiatives to expedite the review process and provide promising therapy for cancer patients while confirmation of clinical benefit (e.g., survival) is ongoing [22]. The FDA’s science-based approach [9, 10] encourages early phase, proof-of-concept trials of botanical products without requiring complete purification of their test substances or identification of all active constituents. This is contingent upon addressing significant quality- and safety-related questions posed by the Agency. For instance, sponsors may provide prior safe human experience, either as traditional medicines or through marketing experience of dietary supplements. If no known human safety concerns are identified, INDs may be allowed to proceed with clinical investigations without animal toxicity studies. This approach provides earlier potential options for patients with terminal diseases.

As of December 2020, about one third (82 out of 254) of the botanical INDs remain active at various clinical trial phases for the management of cancers. For these INDs, the investigations are ongoing, and the botanicals could be developed as new drugs if supported by pivotal clinical trials demonstrating their safety and efficacy, their benefits outweighing the risks, proper product labeling (package insert), adequate CMC, and detailed information on BRM. Successfully approved botanical drugs by FDA are publicly available at Drugs@FDA [23, 24] and DailyMed [25]. Although resources such as ClinicalTrials.gov [26] and institute-sponsored clinical trials [27, 28] including one managed by the National Cancer Institute (NCI) of the NIH [5], provide information on complementary and alternative medicines, there is no published regulatory analysis of botanical INDs submitted to FDA. To the best of our knowledge, our work represents the first in-depth analysis of FDA-evaluated botanical INDs with oncologic indications.

## Clinical Trial Phases and Primary Purpose of Oncologic Botanical INDs

Drug development is a stepwise process and is usually divided into four stages: discovery, preclinical studies in experimental model systems, investigational clinical trials (phase 1–3), and market approval. Botanical drugs derived from plants have been used as traditional medicines for hundreds of years and some of the ingredients have been marketed as dietary supplements in the US based on prior human experience. Their research and development generally start with early phase clinical trials (e.g., phase 1 or 2) as proof-of-concept studies to assess their safety, tolerability, and effectiveness. Our analysis of 254 botanical INDs submitted for oncologic indications showed that there were currently 82 active oncologic botanical INDs, which resulted in 34% (phase 1) and 30% (phase 2) (Fig. 1). Overall, the probability of success of oncological botanical INDs seemed lower than that for conventional INDs, where recently published data [29] showed success rates of 66% and 49% for INDs in phase 1 and 2, respectively. It may be noteworthy that while 24% of oncologic botanical INDs were submitted to the FDA as commercial application INDs and were often sponsored by pharmaceutical manufacturers, who intended to use the data to develop the drugs for the market, over three quarters (76%, data not shown) of clinical investigators submitted research application INDs. We also analyzed the primary purpose of oncologic botanical INDs based on five criteria from the NIH ClinicalTrials.gov protocol registration data elements: basic research, treatment, supportive care, prevention, and diagnostic application [16]. The data elements define the basic research as interventions for examining the basic mechanism of action. Plants produce many phytochemicals and some phytochemicals (e.g., sesquiterpenes, flavonoids, alkaloids, polyphenol) can affect diverse molecular targets and signaling pathways [30] frequently associated with potential carcinogens [31]. Botanical drug research has been valuable for the discovery and development of biologically active, primarily single-molecule based, novel chemotherapies, such as vinblastine, vincristine, teniposide, paclitaxel and camptothecin [32]. Botanical mixtures isolated from more than 3,000 plant species, each containing many active compounds [33] are also being tested in clinical trials to examine basic mechanisms of pro-cancer, mixed, or complementary anti-cancer effects with potentially low-toxicity [34]. In addition, the presence of multiple compounds in a botanical extract are shown to reduce side effects from other therapies [35, 36]. As shown in Figs. 2 and 38% of the botanical INDs were submitted for basic research (e.g., study of tumor markers) as the primary purpose of their clinical trials with most of these trials being in phase 1 (90%, data not shown). The phase 2 and/or phase 3 trials were submitted to investigate both safety and efficacy of botanical products for the treatment (26%, e.g., increased time to progression to more advanced cancer stages), supportive care (20%, e.g., minimized symptoms associated with chemotherapy), or prevention (14%, e.g., reduced recurrence of cancer) in cancer management (Fig. 2).

## Tumor Types for Botanical INDs with Oncological Indications

Our analysis of specific tumor types for the oncologic botanical INDs showed that investigation of solid tumors (181 out of 254, 71%), including prostate, breast, lung, colon/rectal, and hepatic cancers, were the most frequent tumor types. This result was not surprising since these cancer sites account for nearly 50% of all new cancer cases in the US [37]. Among hematologic malignancies (22 out of 254, 9%), myeloma and leukemia were the most-studied, followed by myelosuppression, unspecified malignancies, B-cell malignancies, hemoglobinopathies, lymphoma, lymphoproliferative syndrome, and macroglobulinemia. Overall, our profiling of specific tumor types for oncologic botanical INDs appeared to be in line with the reported rates of new cancers in the US in 2020 [38]. 

## Future Perspectives on Challenge and Opportunity in Oncologic Botanical Products

The regulatory pathways and clinical trials for botanical products in cancer management continue to face several challenges. These include insufficient information on botanical raw materials, which often leads to trial holds or withdrawals in early-phase studies; the inherent variation and complexity of botanical components; and the rigorous evidence-based requirements necessary for drug approval, which can present hurdles in late-phase trials.

Despite these challenges, there has been a steady increase in botanical IND submissions over the past four decades, particularly in cancer management, as analyzed in this paper. Building on the scientific and regulatory insights gained from the successful approval of four botanical drugs in the U.S., the FDA remains committed to a science-based, “totality-of-the-evidence” approach. This strategy aims to incentivize critical research and drug development in oncologic botanical products, provide regulatory flexibility in early-phase clinical trials, and ensure that marketed batches remain therapeutically consistent with those used during premarket clinical development.

## Conclusion

There were over 80 active botanical oncologic INDs received by the FDA between March 1984 and December 2020. Although most of them were in early phase trials (phase 1 and 2) for a variety of tumor types, there were also a handful of products in late-stage trials (phase 3). Our first in-depth assessment of FDA-evaluated botanical INDs with oncologic indications shows that there are many promising active oncologic botanical INDs in the regulatory pipeline for basic scientific research, treatment of various cancers, supportive care for cancer patients, and cancer prevention despite regulatory and scientific challenges.

## Data Availability

No datasets were generated or analysed during the current study.
